# The Dietary Total-Fat Content Affects the In Vivo Circulating C15:0 and C17:0 Fatty Acid Levels Independently

**DOI:** 10.3390/nu10111646

**Published:** 2018-11-03

**Authors:** Benjamin Jenkins, Manar Aoun, Christine Feillet-Coudray, Charles Coudray, Martin Ronis, Albert Koulman

**Affiliations:** 1 NIHR Core Metabolomics and Lipidomics Laboratory, Wellcome Trust-MRC Institute of Metabolic Science, Addenbrooke’s Hospital, University of Cambridge, Cambridge CB2 0QQ, UK; bjj25@medschl.cam.ac.uk; 2 DMEM, INRA, Univ. Montpellier, 34060 Montpellier, France; manar.aoun@gmail.com (M.A.); christine.coudray@inra.fr (C.F.-C.); charles.coudray@inra.fr (C.C.); 3 College of Medicine, Department of Pharmacology & Experimental Therapeutics, Louisiana State University Health Sciences Centre, 1901 Perdido Str., New Orleans, LA 70112, USA; mronis@lsuhsc.edu

**Keywords:** biomarkers, dietary total-fat, pentadecanoic acid, 15:0, heptadecanoic acid, 17:0, odd chain fatty acids

## Abstract

Pentadecanoic acid (C15:0) and heptadecanoic acid (C17:0) have been described as dietary biomarkers of dairy-fat consumption, with varying degrees of reliability between studies. It remains unclear how the total amount of dietary fat, representing one of the main confounding factors in these biomarker investigations, affects C15:0 and C17:0 circulating concentrations independent of their relative intake. Additionally, it is not clear how changes in the dietary total-fat affects other fatty acids in circulation. Through two dietary studies with different total-fat levels but maintaining identical fatty acid compositions, we were able to see how the dietary total-fat affects the fatty acids in circulation. We saw that there was a statistically significant, proportionate, and robust decrease in the endogenous C15:0 levels with an increase in dietary total-fat. However, there was no significant change in the circulating C17:0 concentrations as the total-fat increased. To conclude, the dietary total-fat content and fat-type have a very complex influence on the relative compositions of circulating fatty acids, which are independent of the actual dietary fatty acid composition. Knowing how to manipulate circulating C15:0 and C17:0 concentrations is far-reaching in nutritional/pathological research as they highlight a dietary route to attenuate the development of metabolic disease (both by reducing risk and improving prognosis).

## 1. Introduction

Odd chain fatty acids, specifically pentadecanoic acid (C15:0) and heptadecanoic acid (C17:0), have been described as biomarkers of dairy consumption [1]. However, there are varying degrees of reliability for each fatty acid [2,3,4,5]. Within large observational human studies, both of these fatty acids correlate moderately with their individual intake; which is usually attributed to the intake of dairy fat containing products, such as milk and butter [6]. However, within strictly controlled animal models, there are significant differences in the correlation for the individual odd chain fatty acid between dietary intake and their circulating levels. Within animal models, C15:0 behaves as a direct concentration biomarker of intake, with no notable variation caused by metabolism or endogenous lipogenesis. In contrast, there is convincing evidence showing that C17:0 does not directly relate to dietary intake. Instead, C17:0 endogenous biosynthesis has a significant influence on its circulating levels [2,7]. Furthermore, the literature suggests that C17:0 may have a significant pathophysiological relevance and could be subject to in vivo homeostasis. Whether or not these fatty acids relate to dairy intake, their circulating levels show a significant association with a reduced risk of cardiometabolic diseases [8,9].

Investigating biomarkers within human populations is difficult due to many confounding factors. One typical dietary factor, such as the participant’s total-fat intake, can vary hugely across a study population [10]; it remains unclear if this independently affects the circulating fatty acid levels. For instance, the effect of the total-fat intake in relation to the total energy intake on circulating odd chain fatty acids (C15:0 and C17:0) biomarkers has not been investigated.

The aim of this study was to investigate the influence of the total-fat intake (relative to total energy intake (% total-fat)) on the circulating C15:0 and C17:0 concentrations, independent of the dietary C15:0 and C17:0 composition. The current thought is that both C15:0 and C17:0 are homologous dietary biomarkers (supposed to be independent of endogenous metabolic processes). Therefore, both C15:0 and C17:0 in vivo should reliably correlate with their dietary compositions irrespective of any other dietary factors, such as the dietary total-fat content.

For this project, we decided to make use of samples from existing studies that could provide evidence to achieve the aim of this work. In our opinion, this is a more ethical approach than to start new a study demanding the use of more animals. The two studies we selected included a three-tier-diet dose-response-type study [11] to investigate whether there is a linear effect on C15:0 and C17:0 by changing the total-fat content, and a two-tier-diet with different fat sources [12] to investigate if any change in C15:0 or C17:0 due to the total-fat levels is consistent across different fat sources (i.e., whether there sources of fat that may promote optimal C15:0 or C17:0 levels). The fact that these studies use different lines of rats, different diet formulations, and different time points contributes to the robustness of any reproduced results.

## 2. Materials and Methods

### 2.1. Study One—Three-Tier-Diet Dose-Response

The setup of this study has been previously described in detail [11]. In short, male Sprague-Dawley rats (*n* = 6–7 per group) were housed under standard conditions. Rats had an intragastric cannula surgically inserted seven days before being separated into three groups, with each group receiving an experimental diet with either 5%, 35%, or 70% total-fat content (% energy); fat was isocalorically substituted for carbohydrate calories, and protein, vitamin, and mineral contents were identical in all diets. The animals had *ad libitum* access to water throughout the study. Blood was collected and processed into serum after 21 days on the experimental diets. All samples were stored at −80 °C until being analysed. Animals were housed in an Association for Assessment and Accreditation of Laboratory Animal Care (AAALAC) approved animal facility. Animal maintenance and experimental treatments were conducted in accordance with the ethical guidelines for animal research and were approved by the Institutional Animal Care and Use Committee at the University of Arkansas for Medical Sciences.

### 2.2. Study Two—Two-Tier-Diet And Dietary Fat Source

The setup of this study has been previously described in detail [12]. In short, six-week old male Wistar rats (*n* = 6–8 per group) were housed under standard conditions. The rats were separated into six groups and fed one of six experimental diets made up of three different fatty acid compositions (described as basal-, lard-, and fish oil-based diets) at two different total-fat levels (11% and 51%; % energy). The rats had *ad libitum* access to demineralised water and food. After twelve weeks, the animals were euthanized with pentobarbital, and blood was collected by puncturing the abdominal vein with a heparinised syringe and then centrifuged at 1000 *g* for 10 min at 4 °C to obtain plasma. All samples were stored at −80 °C until being analysed. The INRA (INRA: Institut national de la recherche agronomique) institutional guidelines for the care and use of laboratory animals were followed, and all experimental procedures were approved by the local ethics committee in Montpellier, France (reference CEEALR-11 009).

### 2.3. Analytical Materials

Chemicals were obtained from Sigma-Aldrich (Sigma-Aldrich Company Ltd., Dorset, UK). All solvents were of HPLC (HPLC: high performance liquid chromatography) grade.

### 2.4. Fatty Acid Methyl Ester Preparation

Samples were extracted using the chloroform: methanol: water extraction as previously described by Folch et al. [13]. Briefly, chloroform: methanol solution (2:1, 1 mL) was added to 100 µL of plasma/serum. The samples were then vortexed and sonicated for 15 min. Water (400 µL) was added to each sample, followed by further vortexing, sonication (15 min), and additional vortexing to ensure complete recovery. Samples were centrifuged (~20,000 rpm, 5 min) and the resulting aqueous and organic layers were separated and dried under a gentle stream of nitrogen. For the analysis of total fatty acids, the samples were derivatised as described previously [2]. To the dried organic extracts, boron trifluoride in methanol (14%, 125 µL), chloroform: methanol (1:1, 100 µL), and internal standard tridecanoic acid-_d25_ in chloroform (100 µL, 200 µmol) were added. The mixture was thoroughly vortexed, sonicated (30 min), and vortexed a further time to ensure that there was no undissolved material. Samples were heated to 80 °C for 90 min to commence the derivatisation process. After the samples had cooled, water (300 µL) and hexane (600 µL) were added. The samples were vortexed and the organic layer separated into glass vials, blown down to dryness under nitrogen, and finally reconstituted in 200 µL of hexane, ready for gas chromatography separation with mass spectrometry detection (GC-MS) analysis.

### 2.5. Gas Chromatography Mass Spectrometry Parameters

Gas chromatography separation was achieved using a 6890N/5973 Agilent GC-MS system (Agilent Technologies, California, USA) with a HP-88, 30 m capillary column, with a 0.25 mm internal diameter and a 0.2 µm film thickness (Agilent 112-8837). The inlet temperature was 250 °C. A total oven gradient over 26 min from 120 °C to 210 °C (initial temperature 120 °C hold of 1 min, temperature increase of 10 °C per minute to 170 °C followed by a hold of 6 min, then temperature increase of 3 °C per minute to 210 °C followed by a hold of 1 min) with a 5:1 split was employed. Full scan mass spectrometry detection started after a delay of 2 min (mass range from 60–400 Da, transfer line temperature of 280 °C, MS-source temperature of 230 °C, MS-quadrupole temperature of 150 °C). Peaks were integrated using GC/MSD ChemStation (Agilent Technologies, California, USA) and signal intensities were normalized to the internal standard.

### 2.6. Statistical Analysis

Homoscedastic *t*-tests were used between two unrelated equal variance groups to identify any significance/non-significance between them two groups. For the homoscedastic *t*-tests, a value of *p* ≤ 0.05 was considered statistically significant.

## 3. Results

**Figure 1:**
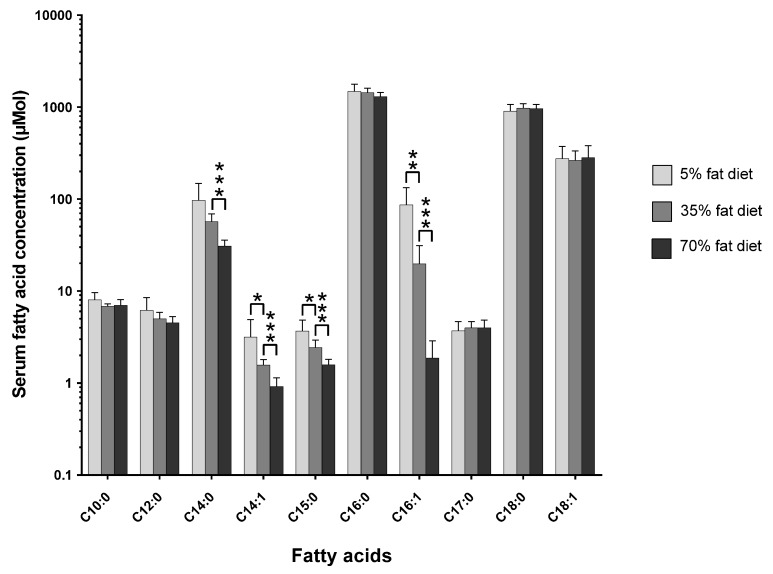
The effect of changing the amount of total-fat within the diet from 5% to 35% to 70% (% energy) on the serum fatty acid concentration (µmol) whilst maintaining identical dietary fatty acid compositions across the three diets (*n* = 6–7 per group). This is to see if the amount of total-fat within the diet influences the serum fatty acid concentration independent of the actual dietary fatty acid composition. The serum samples were analysed by gas chromatography separation with mass spectrometry detection. The significance of the difference between each group is shown by the *p*-value star system, where *p* ≤ 0.05 was considered statistically significant (*p* < 0.05 = *, *p* < 0.01 = **, *p* < 0.001 = ***). Error bars represent ± standard error of the mean.

The pathophysiological effects of the three-tier-diet dose-response study have been previously described in detail [11]. However, the effect of changing the dietary total-fat content from 5% to 35% to 70% on the circulating C15:0 and C17:0 concentrations is shown in Figure 1 (Figure S1 shows the mol % changes; Table S1 and Table S2 show the tabulated data for mol % and µmol, respectively).

As shown in the figure above (see Figure 1), the concentration of C15:0 decreases as the dietary total-fat content increases (*p* < 0.05); a ~30% increase in dietary total-fat resulted in a ~35% decrease in circulating C15:0 concentration. The C17:0 concentration slightly increases, although statistically non-significantly.

**Figure 2:**
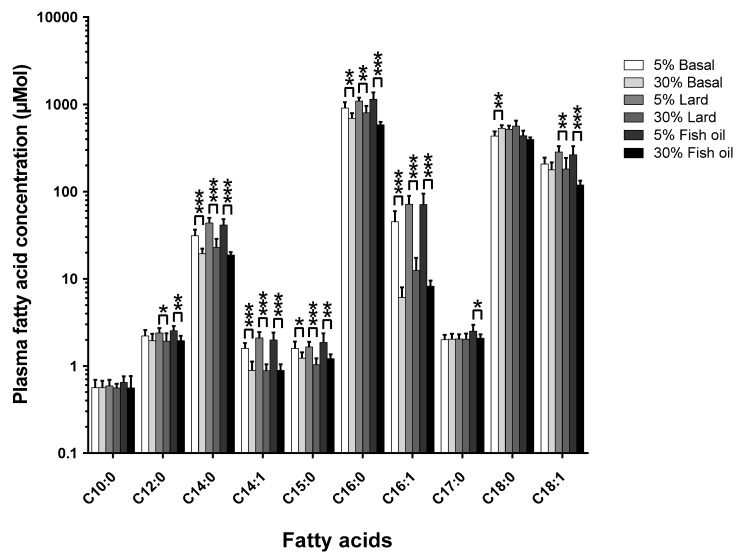
The effect of changing the amount of total-fat within the diet (% energy) from 11% to 51% on the plasma fatty acid concentration (µmol) across three different formulations of dietary fat (Basal, Lard, and Fish oil) (*n* = 6–8 per group). This is to see if the amount of total-fat within the diet influences the plasma fatty acid concentration independent of the dietary fatty acid composition. The plasma samples were analysed by gas chromatography separation with mass spectrometry detection. The significance of the difference between each group is shown by the *p*-value star system, where *p* ≤ 0.05 was considered significant (*p* < 0.05 = *, *p* < 0.01 = **, *p* < 0.001 = ***). Error bars represent ± standard error of the mean.

The pathophysiological results of the two-tier-diet and dietary fat source study have been previously described in detail [12]. The effects of changing the diet’s total-fat content across the three different formulations of fat are shown in Figure 2 (Figure S2 shows the mol % changes; Table S1 and Table S2 show the tabulated data for mol % and µmol, respectively).

The amount of dietary total-fat (as a percentage of total energy) has a significant effect on the concentrations of circulating fatty acids. This correlation in study one (see Figure 1) was confirmed by study two (see Figure 2), where there was a ~40% increase in dietary total-fat content which resulted in a ~30% decrease in the circulating C15:0. On the contrary, there was no significant change in the circulating C17:0 concentrations (*p* > 0.05) for the basal fat and the lard fat-based diets; this agrees with study one (see Figure 1). Interestingly, there was a significant ~16% decrease seen in the circulating C17:0 in the fish oil diet group (*p* < 0.05) within study two (see Figure 2) as the dietary total-fat content increased from 11% to 51%.

The dietary fatty acid compositions were identical between each experimental diet and therefore, any changes in the fatty acid concentrations in vivo were due to the change in the dietary total-fat content.

Interestingly, these results confirm previous studies on even chain fatty acids, where increasing the dietary total-fat content independently effects circulating fatty acid levels [14].

## 4. Discussion

To our knowledge, this is the first report that shows the effects of varying the dietary total-fat (as a percentage of total energy) on the circulating C15:0 and C17:0 fatty acids concentrations whilst maintaining the actual dietary C15:0 and C17:0 fatty acid composition (mol %).

As shown in study one (see Figure 1), there was a statistically significant (*p* < 0.05), proportionate, and robust decrease in the endogenous C15:0 levels with an increase in dietary total-fat content from 5% to 35% to 70% (% energy). However, there was no statistically significant change in the endogenous C17:0 concentrations. The inverse correlation seen in C15:0 suggests there could be a decrease in the efficiency of intestinal absorption as the dietary total-fat content increases. The correlations with the dietary total-fat content show that C15:0 and C17:0 are not homologous with each other and that they must be discriminated from one another when being studied; either because they originate from different sources or because they could be biologically compartmentalised. These absolute concentration relationships show that C15:0 and C17:0 are changing with varying dietary total-fat contents and are not artificially created due to changes in endogenous lipogenesis (as seen in relative compositions; mol %).

In the literature, it is shown that C15:0 and C17:0 may originate from different sources; C15:0 appears to come directly from the diet, while C17:0 is also significantly endogenously biosynthesized [2,7]. The results presented here support a dietary origin for C15:0, since the in vivo C15:0 compositions decrease with an increase in dietary total-fat. This data was supported by our second study comparing circulating fatty acids across two total-fat levels (11% and 51%) and three different diet formulations (basal fat-, lard fat-, and fish oil-based) (see Figure 2). For C17:0, the basal fat and lard fat diets each saw no statistically significant change in the in vivo C17:0 concentrations with an increase in the dietary total-fat content. However, the fish oil-based diet resulted in a statistically significant decrease in the in vivo C17:0 concentrations (~16%, *p* < 0.05), which further suggests C17:0 endogenous biosynthesis since it has been shown that n-3 poly-unsaturated fatty acids significantly decrease *Hacl1* gene expression [15]; shown to be involved in C17:0 biosynthesis [7].

Many epidemiological studies have shown that there is an inverse association between the relative compositions/concentration of odd chain fatty acids and metabolic disease risk [8,9]. From work in animal models, we showed that this relationship could be causal, at least for C17:0; a reduction in C17:0 could be significant in the development of pathology [2]. It is therefore essential to understand how diet will affect the relative compositions of these two odd chain fatty acids; C15:0 and C17:0. The findings from these studies highlight the need for strictly controlled confirmation of biomarkers, especially when they originate from large human studies, due to the inherent complexity of the data sets involved. The effects of the dietary total-fat can be further complicated by processes like de novo lipogenesis [16] (influenced by non-fat dietary components, such as carbohydrates or ethanol [17] (see Table S3: The circulating fatty acid compositions (mol %) changes between an ethanol treated group and their associated control group)).

Limitations of this study: The work presented in this manuscript shows that C15:0 and C17:0 are not homologous to one another; however, further work is needed to comprehensively investigate all the contributions to the circulating levels, including different mechanisms of endogenous biosynthesis along with direct dietary contributions.

## 5. Conclusions

From our results, we conclude that the dietary total-fat content and fat-type composition have a very complex influence on the relative concentration of odd chain fatty acids in the circulation, which behaves profoundly differently between C15:0 and C17:0. It is seen that an increase in dietary total-fat significantly independently reduces the circulating levels of C15:0. Furthermore, a fish oil-based diet proves to be noteworthy as it appears to decrease both C15:0 and C17:0. The importance of these findings are far-reaching in nutrition and pathophysiology research as they highlight a possible dietary/nutritional route to attenuate the development of metabolic disease; the impact of these results in humans still needs to be further investigated.

## Supplementary Materials

The following are available online at http://www.mdpi.com/2072-6643/10/11/1646/s1, Figure S1: The circulating fatty acid relative composition (mol %) change in response to the three experimental diets. Figure S2: The circulating fatty acid relative composition (mol %) change in response to the three experimental diets with two different fat formulations. Table S1: The circulating fatty acid composition (mol %) changes with an increase in dietary total-fat. Table S2: The circulating fatty acid concentrations (µmol) changes with an increase in dietary total-fat. Table S3: The circulating fatty acid compositions (mol %) changes between an ethanol treated group and their associated control group.
