# Supplementary material for: The Dietary Total-Fat Content Affects the In Vivo Circulating C15:0 and C17:0 Fatty Acid Levels Independently
Source: Nutrients. 2018 Nov 3;10(11):1646. doi: 10.3390/nu10111646 (PMC6266905; doi:10.3390/nu10111646)
Supplement: Supplementary file 1 [file nutrients-10-01646-s001.pdf]

SUPPLEMENTARY INFORMATION:

Figure S1: The circulating fatty acid relative composition (mol %) change in response to the three experimental diets.

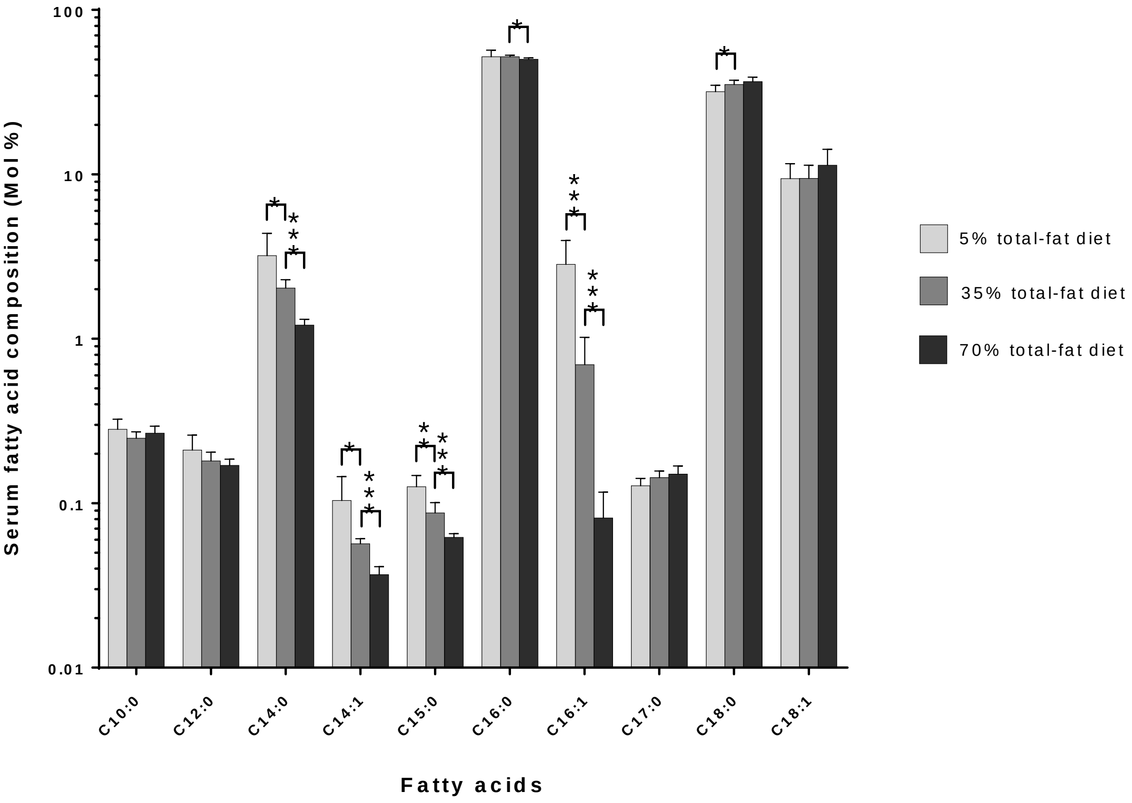

Figure S1; The effect of changing the amount of total-fat within the diet from 5% to 35% to 70% (% total energy) on the serum fatty acid relative compositions (mol %) whilst maintaining identical dietary fatty acid compositions across the three diets (n = 6-7 per group). This is to see if the amount of fat within the diet influences the serum fatty acid relative compositions independent of the actual dietary fatty acid composition. The serum samples were analysed by gas chromatography separation with mass spectrometry detection. The significance of the difference between each group is shown by the p-value star system; where $p \leq 0.05$ was considered statistically significant ($p < 0.05$ = *, $p < 0.01$ = **, $p < 0.001$ = ***). Error bars represent ± standard error of the mean.

Figure S2: The circulating fatty acid relative composition (mol %) change in response to the three experimental diets with two different fat formulations.

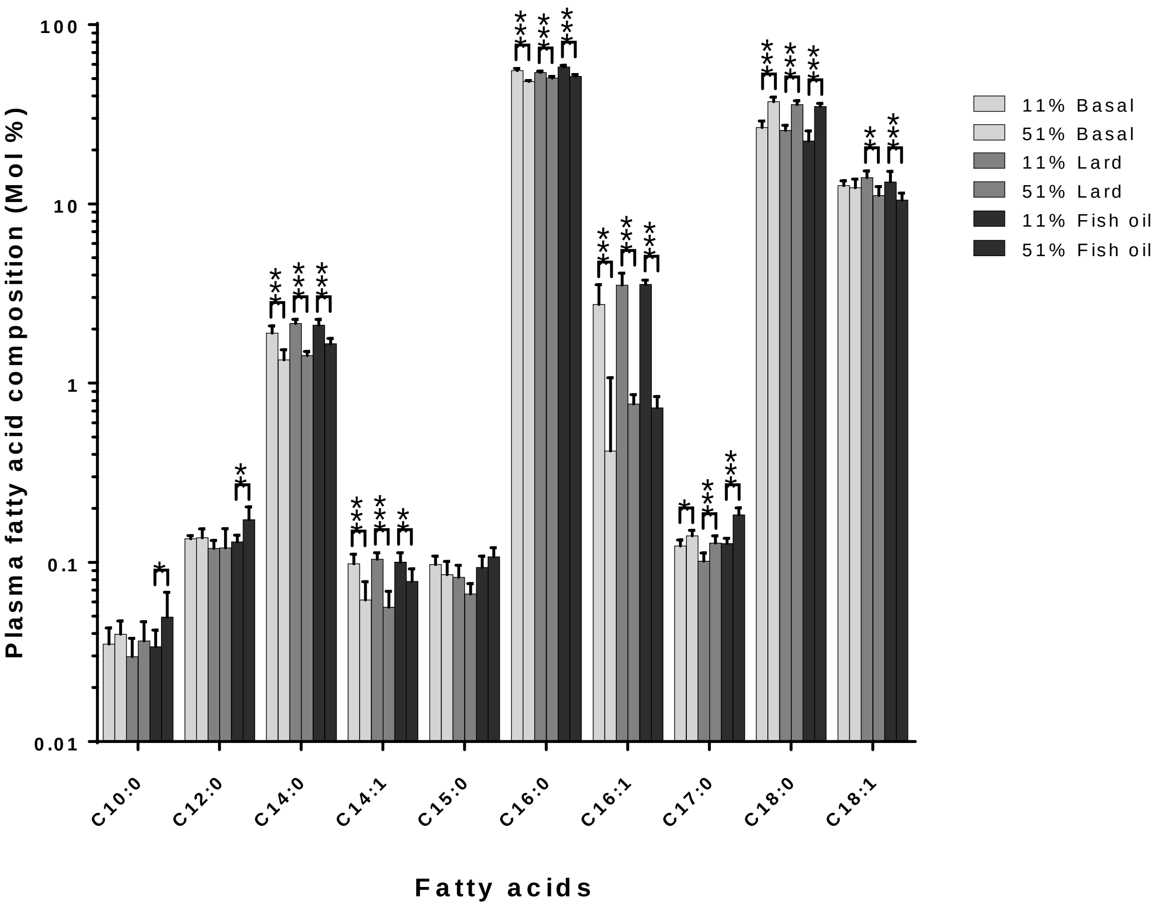

Figure S2; The effect of changing the amount of total-fat within the diet (% energy) from 11% to 51% on the plasma fatty acid relative compositions (mol %) across three different formulations of dietary fat (Basal, Lard, and Fish oil) (n = 6-8 per group). This is to see if the amount of total-fat within the diet influences the plasma fatty acid composition independent of the dietary fatty acid composition. The plasma samples were analysed by gas chromatography separation with mass spectrometry detection. The significance of the difference between each group is shown by the p-value star system; where $p \leq 0.05$ was considered significant ($p < 0.05$ = *, $p < 0.01$ = **, $p < 0.001$ = ***). Error bars represent ± standard error of the mean.

Table S1: The circulating fatty acid composition (mol %) changes with an increase in dietary total-fat.

Table S1; The effect of changing the amount of total-fat within the diet on the circulating fatty acid composition (mol %) whilst maintaining identical dietary fatty acid compositions across the experimental diets (n = 6-8 per group). This is to see if the amount of total-fat within the diet influences the circulating fatty acid composition independent of the actual dietary fatty acid composition. The samples were analysed by gas chromatography separation with mass spectrometry detection. Values ± standard error of the mean. * denotes statistically significant change.

Study	Group	n=	C10:0	C12:0	C14:0	C14:1	C15:0	C16:0	C16:1	C17:0	C18:0	C18:1
1	5%	7	0.282 ± 0.043	0.211 ± 0.049	3.205 ± 1.169	0.104 ± 0.041	0.126 ± 0.022	51.870 ± 5.007	2.837 ± 1.126	0.128 ± 0.014	31.814 ± 3.015	9.424 ± 2.173
1	35%	7	0.248 ± 0.023	0.181 ± 0.024	2.037 ± 0.250*	0.057 ± 0.004*	0.087 ± 0.013*	51.895 ± 1.106	0.697 ± 0.325*	0.143 ± 0.014	35.218 ± 2.100*	9.437 ± 1.912
1	70%	6	0.267 ± 0.028	0.170 ± 0.015	1.212 ± 0.103*	0.037 ± 0.004*	0.062 ± 0.003*	50.057 ± 1.012*	0.081 ± 0.035*	0.150 ± 0.018	36.601 ± 2.399	11.361 ± 2.850
2	11% Basal	6	0.035 ± 0.008	0.135 ± 0.006	1.903 ± 0.186	0.098 ± 0.013	0.097 ± 0.011	55.499 ± 1.513	2.745 ± 0.796	0.123 ± 0.010	26.693 ± 2.342	12.671 ± 0.805
2	51% Basal	8	0.04 ± 0.007	0.137 ± 0.017	1.348 ± 0.188*	0.062 ± 0.016*	0.085 ± 0.016	48.243 ± 0.549*	0.419 ± 0.652*	0.141 ± 0.010*	37.208 ± 2.234*	12.318 ± 1.446
2	11% Lard	8	0.03 ± 0.008	0.120 ± 0.013	2.155 ± 0.110	0.104 ± 0.009	0.082 ± 0.014	54.100 ± 0.950	3.527 ± 0.584	0.101 ± 0.011	25.746 ± 1.688	14.035 ± 1.249
2	51% Lard	7	0.036 ± 0.01	0.120 ± 0.034	1.426 ± 0.075*	0.056 ± 0.013*	0.067 ± 0.009	50.431 ± 1.075*	0.764 ± 0.099*	0.128 ± 0.012*	35.833 ± 1.837*	11.138 ± 1.347*
2	11% Fish oil	8	0.034 ± 0.008	0.130 ± 0.012	2.106 ± 0.161	0.100 ± 0.013	0.094 ± 0.015	58.158 ± 1.099	3.552 ± 0.202	0.127 ± 0.009	22.452 ± 3.142	13.247 ± 1.967
2	51% Fish oil	8	0.050 ± 0.019*	0.173 ± 0.031*	1.658 ± 0.116*	0.078 ± 0.014*	0.107 ± 0.013	51.510 ± 1.276*	0.728 ± 0.115*	0.184 ± 0.017*	34.99 ± 1.342*	10.522 ± 0.972*

Table S2: The circulating fatty acid concentration (µmol) changes with an increase in dietary total-fat.

Table S2; The effect of changing the amount of total-fat within the diet on the circulating fatty acid concentrations (µmol) whilst maintaining identical dietary fatty acid compositions across the experimental diets (n = 6-8 per group). This is to see if the amount of total-fat within the diet influences the circulating fatty acid composition independent of the actual dietary fatty acid composition. The samples were analysed by gas chromatography separation with mass spectrometry detection. Values ± standard error of the mean. * denotes statistically significant change.

Study	Group	n=	C10:0	C12:0	C14:0	C14:1	C15:0	C16:0	C16:1	C17:0	C18:0	C18:1
1	5%	7	8.003 ± 1.648	6.170 ± 2.301	97.276 ± 51.193	3.154 ± 1.747	3.666 ± 1.165	1480.840 ± 295.240	86.424 ± 47.440	3.698 ± 0.965	906.615 ± 163.796	275.565 ± 100.404
1	35%	7	6.816 ± 0.452	4.998 ± 0.869	56.743 ± 12.349	1.569 ± 0.233*	2.431 ± 0.500*	1435.370 ± 169.906	19.780 ± 11.358*	3.979 ± 0.666	973.550 ± 121.588	264.571 ± 71.300
1	70%	6	7.023 ± 1.071	4.506 ± 0.785	30.966 ± 4.917*	0.918 ± 0.222*	1.580 ± 0.233*	1302.439 ± 147.839	1.871 ± 1.012*	3.986 ± 0.835	964.247 ± 107.531	283.341 ± 97.865
2	11% Basal	6	0.563 ± 0.131	2.209 ± 0.365	31.057 ± 5.577	1.586 ± 0.247	1.583 ± 0.317	905.889 ± 149.429	45.063 ± 14.474	1.998 ± 0.277	432.443 ± 58.813	207.035 ± 38.358
2	51% Basal	8	0.564 ± 0.112	1.941 ± 0.396	19.373 ± 2.711*	0.888 ± 0.235*	1.228 ± 0.202*	694.203 ± 96.351*	6.091 ± 1.863*	2.022 ± 0.319	532.157 ± 44.059*	178.396 ± 37.623
2	11% Lard	8	0.590 ± 0.100	2.399 ± 0.311	43.536 ± 6.532	2.091 ± 0.352	1.643 ± 0.252	1090.292 ± 107.886*	71.709 ± 17.457	2.040 ± 0.264	516.889 ± 51.562	283.970 ± 48.046
2	51% Lard	7	0.561 ± 0.063	1.920 ± 0.444*	22.852 ± 5.751*	0.878 ± 0.167*	1.035 ± 0.193*	803.398 ± 156.435	12.459 ± 4.957*	2.029 ± 0.324	565.145 ± 83.176	181.141 ± 62.088*
2	11% Fish oil	8	0.648 ± 0.110	2.528 ± 0.332	41.297 ± 7.000	1.980 ± 0.439	1.853 ± 0.502	1146.430 ± 225.918*	70.903 ± 23.544	2.498 ± 0.471	437.915 ± 59.997	263.028 ± 67.454
2	51% Fish oil	8	0.559 ± 0.209	1.941 ± 0.264*	18.718 ± 1.388*	0.885 ± 0.156*	1.210 ± 0.151*	582.713 ± 46.481	8.208 ± 1.304*	2.083 ± 0.226*	395.231 ± 23.734	119.017 ± 14.294*

Table S3: The circulating fatty acid composition (mol %) changes between an ethanol treated group and an associated control group.

Table S3; The serum fatty acid composition (mol %) of the ethanol treated Sprague-Dawley rats (EtOH) and the associated control group (Control) measured by gas chromatography separation with mass spectrometry detection following an intragastric cannula feeding experiment where either group received 35% corn oil or 35% corn oil with 39% ethanol (as percentage of total energy, ethanol was isocalorically substituted for carbohydrate calories; additionally, protein, vitamin, and mineral contents were identical in all diets). The significance of the difference between the two groups is shown by the p-value. A value of $p \leq 0.05$ was considered significant. Results are shown with ± standard error of the mean. (n = 7 per group; male).

	Control group (mol %)	EtOH group (mol %)	p-value
C10:0	0.202 ± 0.010	0.223 ± 0.017	>0.1
C12:0	0.147 ± 0.006	0.149 ± 0.006	>0.1
C14:0	1.652 ± 0.071	1.351 ± 0.042	0.003
C14:1	0.046 ± 0.002	0.039 ± 0.002	0.020
C15:0	0.071 ± 0.003	0.058 ± 0.003	0.014
C16:0	42.173 ± 0.855	39.733 ± 0.560	0.034
C16:1	0.565 ± 0.095	0.343 ± 0.063	0.077
C17:0	0.116 ± 0.004	0.112 ± 0.004	>0.1
C18:0	28.657 ± 1.014	29.880 ± 1.012	>0.1
C18:1	7.615 ± 0.471	7.324 ± 0.482	>0.1
C18:2	18.755 ± 1.271	20.787 ± 0.901	>0.1
